# *Trueperella pyogenes*–Associated Acute Pneumonia in Korean Native Calves

**DOI:** 10.1002/vms3.70913

**Published:** 2026-03-27

**Authors:** Min‐Jeong Ji, Youngjun Kim, Byoung‐Soo Kim, Jongho Kim, Kyoung‐Seong Choi

**Affiliations:** ^1^ Department of Animal Science and Biotechnology, College of Ecology and Environmental Science Kyungpook National University Sangju Republic of Korea; ^2^ Hanwoo (Korean Indigenous Cattle) Genetic Improvement Center Nonghyup Agribusiness Group Inc. Seosan Republic of Korea; ^3^ Yeongkwang Veterinary Clinic Yeonggwang Republic of Korea; ^4^ Animal Disease Diagnostic Division Animal and Plant Quarantine Agency Gimcheon Republic of Korea

**Keywords:** antibiotic resistance gene, bovine respiratory disease, *Trueperella pyogenes*

## Abstract

Bovine respiratory disease (BRD) is a leading cause of morbidity and mortality in the cattle industry worldwide, resulting in substantial economic losses. *Trueperella pyogenes* has been recognized as an opportunistic pathogen; however, its role as a primary aetiologic agent of BRD remains poorly defined. Four Korean native calves developed respiratory signs, including coughing, nasal discharge and pneumonia, and died within 1–2 days after onset. Postmortem examination revealed characteristic gross pulmonary lesions. PCR was performed to detect common BRD pathogens and antimicrobial resistance (AMR) genes. *T. pyogenes* was identified in the lungs of all calves examined. Three calves were infected solely with *T. pyogenes*, whereas one calf was co‐infected with three pathogens (*Histophilus somni*, *Mycoplasma dispar* and *Pasteurella multocida*) as well as *T. pyogenes*. AMR genes, predominantly the macrolide resistance genes *ermX* and *ermB*, were detected in three calves. To the best of our knowledge, this is the first report of *T. pyogenes*‐associated pneumonia and the characterization of AMR genes in Korean native calves. This case indicates that *T. pyogenes* can act as a major pathogen responsible for BRD in calves. These findings highlight the pathogenic potential of *T. pyogenes* and the need for targeted therapeutic approaches to support prudent antimicrobial use in cattle.

## Graphical abstract

*Trueperella pyogenes* can cause a wide range of suppurative infections, including urogenital and mammary infections, liver abscesses and pneumonia. This case report demonstrates *Trueperella pyogenes* as a primary cause of bovine respiratory disease in Korean native calves. Severe pulmonary lesions, multiple virulence factors and antimicrobial resistance genes (*ermB*, *ermX* and *tetW*) were identified. These findings underscore the need for judicious antimicrobial use to prevent the spread of multidrug‐resistant bacteria in cattle populations.

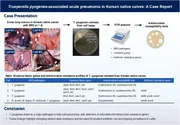

## 1 Introduction

Bovine respiratory disease (BRD) is the second most common disease in neonatal calves after diarrhoea and is a major cause of mortality before and after weaning (Kim et al. 2024). This disease imposes a substantial economic burden on the cattle industry due to mortality, growth retardation, reduced productivity and increased treatment costs, with global losses estimated at approximately $2 billion annually (Wilson et al. 2017). Among the various factors associated with BRD, infectious agents are recognized as the primary contributors to disease onset (Park and Kim 2019).

*Trueperella pyogenes* is an opportunistic pathogen that commonly colonizes the mucosa of the upper respiratory, gastrointestinal and urogenital tracts, as well as the skin of animals. It can cause a wide range of suppurative infections, including urogenital and mammary infections, liver abscesses and pneumonia (Yamamoto et al. 2024). In recent years, the detection rate of *T. pyogenes* has markedly increased in cattle with respiratory diseases, suggesting a close association between this pathogen and BRD (Yamamoto et al. 2024).

Antimicrobial agents are widely used to treat and prevent BRD in cattle. However, excessive or inappropriate use can promote the emergence of antibiotic resistance (Fulton et al. 2009). Macrolides and tetracyclines, in particular, are among the most frequently administered classes for metaphylaxis and therapy in BRD management (Anholt et al. 2017), and resistance to these antibiotics has markedly increased in recent years (Dutta et al. 2021). The rise of antimicrobial resistance (AMR) is now recognized as a critical One Health issue, attracting global attention in both human and veterinary medicine.

Here, we report *T. pyogenes* as a significant pathogen in calves with respiratory disease and describe tetracycline and macrolide resistance genes detected in the isolates.

## 2 Case Presentation

Four Korean native calves presented with severe respiratory distress, characterized by open‐mouth and laboured breathing and were confirmed to have respiratory acidosis associated with increased PCO_2_. On auscultation, distinct crackles and wheezes were detected. All calves were treated with ceftiofur as an antibiotic and the expectorant bromhexine. Aminophylline, diluted in 200 mL of 5% dextrose, was administered as an intravenous bronchodilator infusion over 30 min. Despite treatment, all calves died within 1–2 days after the onset of clinical signs, which included coughing, nasal discharge and pneumonia. Local veterinarians performed necropsies and submitted lung tissues to the Animal Immunology Laboratory at Kyungpook National University for further analysis.

**FIGURE 1:**
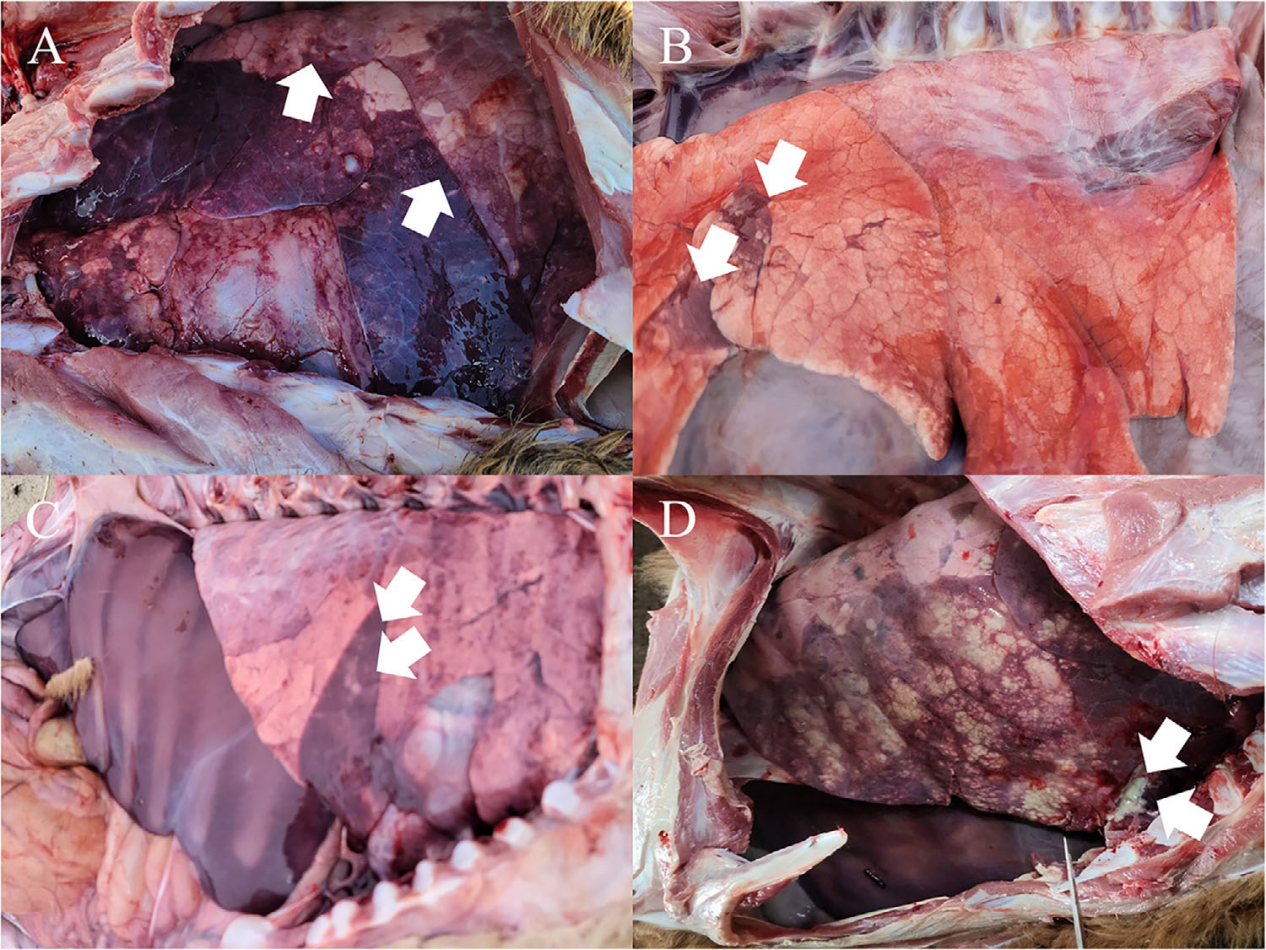
Gross lesions in the lungs from four Korean native calves that died of bovine respiratory disease. (A) severe cranioventral lung lobe consolidations in Calf #1; (B) mild focal lung consolidations in Calf #2; (C) noncollapsed lungs with marked redness and focal lung lobe consolidation in Calf #3; (D) diffuse consolidations of the entire lung lobe with pulmonary abscesses in Calf #4. White arrows indicate lesions.

**FIGURE 2:**
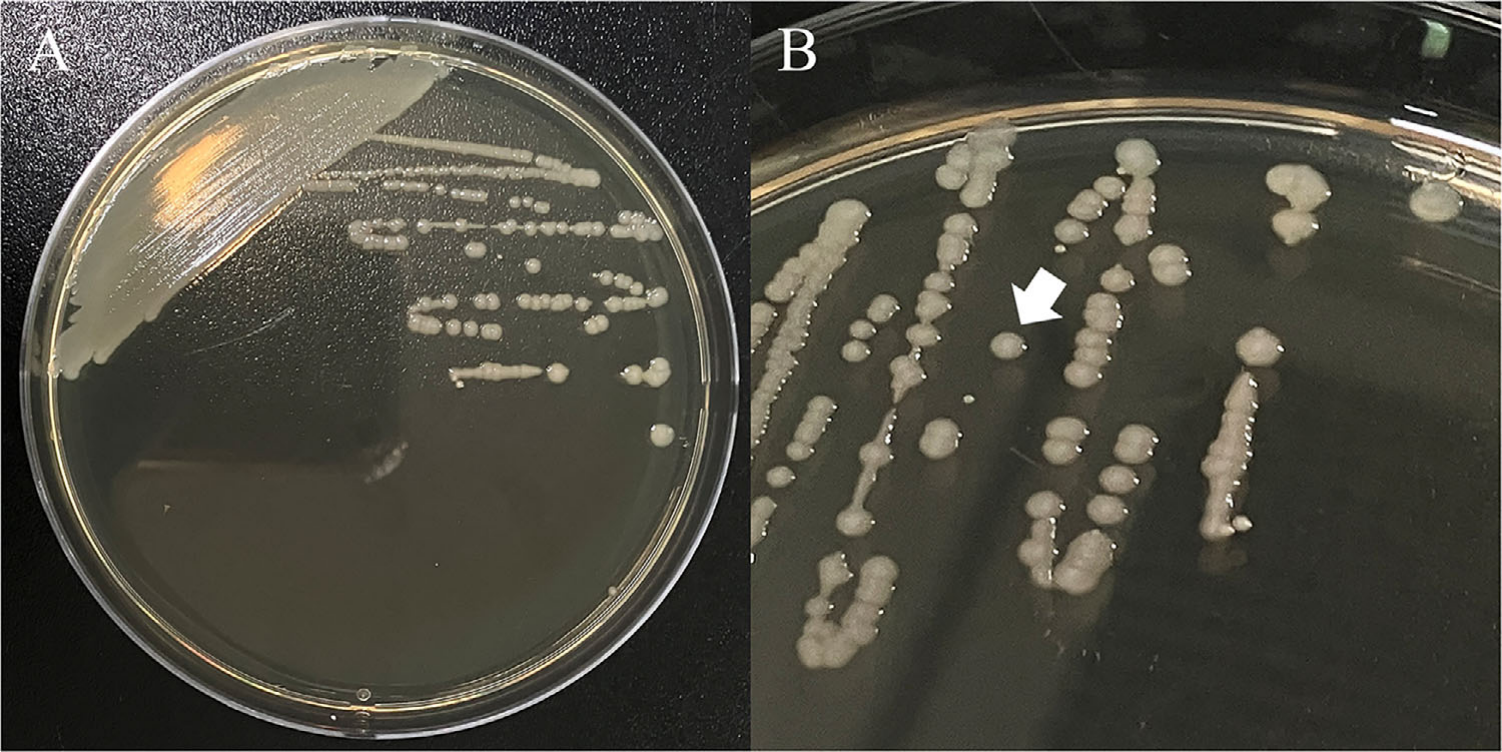
Bacterial colonies of *Trueperella pyogenes* isolated from calf lung tissues. (A) Brain Heart Infusion agar plate showing growth of *T. pyogenes* colonies after 48 h of incubation under facultative anaerobic conditions at 37°C; (B) white, smooth and glistening colonies of *T. pyogenes* as indicated by the white arrows.

All calves had characteristic gross pulmonary lesions. In Calf #1, marked cranioventral consolidation was observed (Figure 1A). Calf #2 exhibited mild focal lung consolidation (Figure 1B). The lung of Calf #3 was noncollapsed and showed marked hyperaemia with focal lobar consolidation (Figure 1C). Calf #4 had diffuse consolidation of all lung lobes with pulmonary abscesses (Figure 1D). Lung tissues, including abscesses, from all four calves were cultured on brain heart infusion agar plates for 48 h at 37°C under facultative anaerobic conditions. Bacterial growth was observed on all plates; notably, all cultures yielded white, smooth, glistening colonies (Figure 2). These colonies were presumptively identified as *T. pyogenes* based on their morphology. No histopathological examination was carried out.

Nucleic acids were extracted from the lungs using the DNeasy Blood & Tissue Kit and RNeasy Mini Kit (Qiagen, Hilden, Germany) according to the manufacturer's instructions. PCR and RT‐PCR were performed to detect major pathogens associated with BRD. First, *Mycoplasma bovis* and *Mycoplasma dispar* were screened by multiplex PCR. *Pasteurella multocida*, *Mannheimia haemolytica* and *T. pyogenes* were identified by a separate multiplex PCR, and *Histophilus somni* was examined using a single‐PCR assay. To assess other respiratory viral infections, bovine herpesvirus‐1 (Aslan et al. 2015), bovine respiratory syncytial virus and bovine parainfluenza virus type 3 (Klima et al. 2014) were also tested. Distilled water was included in each PCR run as a negative control. All PCR products were separated on 1.2% agarose gels and visualized by ethidium bromide staining. No viral pathogens were detected in any of the four calves. PCR analysis consistently identified *T. pyogenes* in lung tissue from all examined calves. Of the four calves, three were infected only with *T. pyogenes*, whereas one calf was co‐infected with *H. somni*, *M. dispar* and *P. multocida*, in addition to *T. pyogenes* (Table 1). The virulence‐gene profile for each calf is summarized in Table 1. All isolates carried the virulence‐associated *plo* gene and the fimbrial genes *fimA* and *fimC*; other virulence factors varied across isolates.

**TABLE 1:** Virulence factor genes detected in *Trueperella pyogenes* isolates from Korean native calves.

Calf	Detected pathogen	Virulence factor genes
1	*Trueperella pyogenes*	*cbpA*, *fimA, fimC, plo*
2	*T. pyogenes*	*cbpA, fimA, fimC, fimG, nanP, nanH, plo*
3	*T. pyogenes*	*fimA, fimC, nanH, plo*
4	*Histophilus somni, Mycoplasma dispar, Pasteurella multocida, T. pyogenes*	*cbpA, fimA, fimC, nanP, plo*

Abbreviations: *cbpA*, collagen‐binding protein A; *fimA*, fimbriae A; *fimC*, fimbriae C; *fimG*, fimbriae G; *nanH*, neuraminidase H; *nanP*, neuraminidase P; *plo*, pyolysin.

Antimicrobial susceptibility testing was performed for isolates from three calves. Testing was not interpreted for Calf #4 because co‐infection with other bacterial pathogens precluded reliable susceptibility results. The tested isolates were resistant to penicillin, oxytetracycline, lincomycin and clindamycin but were susceptible to ciprofloxacin. Susceptibility to other antimicrobial agents varied, including amoxicillin, ceftiofur, florfenicol, enrofloxacin, ofloxacin, erythromycin and streptomycin. We also screened for AMR genes by PCR using specific primers targeting the macrolide resistance genes *ermB* and *ermX* and the tetracycline resistance gene *tetW* (Rezanejad et al. 2019). As shown in Table 2, no AMR genes were detected in Calf #1, whereas the remaining calves carried one or more resistance genes. Among the AMR genes detected, macrolide resistance genes were the most prevalent (Table 2).

**TABLE 2:** Profiles of antimicrobial susceptibility and antimicrobial resistance genes in Korean native calves.

Calf	Antimicrobial susceptibility tests	Antimicrobial resistance genes
1	Erythromycin (R), oxytetracycline (R)	—
2	Erythromycin (I), oxytetracycline (R)	*ermX*
3	Erythromycin (R), oxytetracycline (R)	*tetW, ermB* and *ermX*
4	Not performed	*ermB* and *ermX*

*Note*: —, not detected.

Abbreviations: *ermB*, erythromycin ribosome methylase B; *ermX*, erythromycin ribosome methylase X; I, intermediate; R, resistant; *tetW*, tetracycline‐resistance protein W.

## 3 Discussion

*T. pyogenes* was detected in every calf with respiratory symptoms. Only one calf was co‐infected with other bacterial agents commonly linked to BRD, and those pathogens were absent from the remaining calves. This pattern supports *T. pyogenes* as a major BRD pathogen rather than a purely secondary opportunist. Our results are consistent with prior reports showing frequent detection of *T. pyogenes* in calves with BRD (Antonis et al. 2022; Barroso‐Arévalo et al. 2025; Scott 2013; Zhou et al. 2023). In particular, *T. pyogenes* has been identified as the most commonly isolated bacterium from lung lesions and has been recognized as a major aetiological agent of BRD (Nishi et al. 2025). Several studies have reported that *T. pyogenes* is frequently detected in lesions, such as suppurative or caseonecrotic pneumonia, and is the predominant bacterium among BRD pathogens (Zhou et al. 2023). Although only one calf in this study exhibited pulmonary abscesses, these lesions are consistent with the characteristic suppurative pathology caused by *T. pyogenes*, thereby supporting its role as an aetiological agent of suppurative pneumonia. This is in accordance with previous descriptions of *T. pyogenes*, which is commonly associated with pyogenic infections in livestock (Radostits et al. 2007). Furthermore, recent research has demonstrated a high isolation frequency of *T. pyogenes* in calves with BRD compared to other bacterial agents (Yamamoto et al. 2024). Collectively, these findings suggest that *T. pyogenes* is not merely a secondary pathogen but plays a key role in the onset and pathological progression of BRD in calves. Our results support this observation; the consistent identification of *T. pyogenes* as the dominant agent aligns with the observed pulmonary pathology in the affected calves.

The lungs of the affected calves exhibited cranioventral consolidation, marked redness, noncollapsed lobes, and, in one case, pulmonary abscesses, findings consistent with the typical pathological features of *T. pyogenes*‐induced pneumonia. *T. pyogenes* is recognized as an opportunistic pathogen that causes suppurative pneumonia and various pyogenic infections in many animal species. In ruminants, *T. pyogenes* infection is commonly associated with mastitis, metritis, abscesses and pneumonia (Ribeiro et al. 2015). Bronchopneumonia caused by *T. pyogenes* has been reported in muskox (*Ovibos moschatus*) (Afema et al. 2017), and pneumonia‐associated mortality has been reported in Dall's sheep (*Ovis dalli dalli*) (Jenkins et al. 2007). In sheep, *T. pyogenes* infection is associated with multiple pulmonary abscesses and chronic suppurative bronchopneumonia, with extensive caseous abscesses identified in the cranial lobes of the lungs (Hariharan et al. 2016). Similar pneumonia accompanied by airway inflammation has been documented in swine (Dong et al. 2019). Experimental infection in mice resulted in extensive degeneration of bronchiolar epithelial cells (Qin et al. 2023). Collectively, these findings indicate that *T. pyogenes* can cause respiratory disease in a broad range of hosts, including ruminants, swine and rodents (Dong et al. 2019; Ribeiro et al. 2015). Therefore, the suppurative bronchopneumonia observed in the present cases represents a typical manifestation of *T. pyogenes* infection.

Although *T. pyogenes* is not classically considered a primary respiratory pathogen, evidence increasingly implicates it as an opportunistic or secondary bacterial contributor to BRD, especially in animals weakened by viral infections or other predisposing conditions (Antonis et al. 2022; Eroksuz et al. 2021; Yamamoto et al. 2024; Zhou et al. 2023). Previous studies have reported its association with suppurative infections, including mastitis, abscesses and pneumonia (Ribeiro et al. 2015), underscoring its role in exacerbating clinical severity. However, information on its role in respiratory disease remains scarce in the ROK. The present cases, therefore, constitute the first clinical report identifying *T. pyogenes* as a major aetiologic agent of BRD in Korean native calves. Given its capacity to drive pyogenic inflammation and its frequent association with virulence determinants and AMR, *T. pyogenes* should be regarded as a clinically relevant component of the BRD complex. Diagnostic evaluations for BRD should include targeted testing for *T. pyogenes*.

Antimicrobial susceptibility testing showed uniform resistance of the tested *T. pyogenes* isolates to oxytetracycline. For erythromycin, two isolates were resistant, and one showed intermediate susceptibility. Molecular analysis detected the macrolide‐resistance genes *ermX* (three isolates) and *ermB* (two isolates), and the tetracycline‐resistance gene *tetW* (one isolate). Notably, one isolate carried all three genes (*ermX*, *ermB* and *tetW*), whereas another carried none, indicating heterogeneity in resistance determinants. Despite consistent oxytetracycline resistance, *tetW* was detected in only one isolate. Likewise, the erythromycin phenotypes did not fully correspond to the distribution of macrolide resistance genes. This phenotype–genotype discordance may reflect differences in gene expression, regulatory control or additional resistance mechanisms. Overall, these findings underscore the complexity of AMR in calf‐derived *T. pyogenes* and support combining phenotypic and genotypic data to define resistance profiles accurately. Studies with larger sample sizes are needed to clarify the underlying mechanisms.

Macrolides are commonly used for the treatment and metaphylaxis of BRD (Anholt et al. 2017). Although they are effective in controlling infection and reducing mortality (Abi Younes et al. 2024), their extensive use can promote AMR. In *T. pyogenes*, macrolide resistance is most often driven by *erm* genes encoding 23S rRNA methyltransferases that alter the ribosomal binding site targeted by macrolide–lincosamide–streptogramin B (MLS_B) antibiotics (Marchionatti and Perreten 2023). Among these, *ermB* and *ermX* are frequently detected in *T. pyogenes* and can facilitate horizontal transfer and dissemination of multidrug resistance (Marchionatti and Perreten 2023). The detection of *ermB* and *ermX* in these isolates suggests that macrolide resistance may have emerged under selective pressure from antibiotic use and highlights the potential for dissemination of these genes within the herd.

Tetracyclines are also broadly used in veterinary medicine (Kwiecień et al. 2021). Resistance is commonly mediated by *tet* genes encoding drug efflux or ribosomal protection proteins (Kwiecień et al. 2021). The *tetW* gene is widespread among *T. pyogenes* isolates and can confer multidrug resistance, thereby promoting the emergence of multidrug‐resistant bacteria and the continued dissemination of resistance genes (Zhang et al. 2017). Although *tetW* was identified in only one isolate, the concurrent detection of *ermB*, *ermX* and *tetW* suggests selective pressure from antibiotic use and the potential for dissemination of resistance genes within the herd.

This study had important limitations. First, the small sample size and absence of histopathology prevented us from directly linking pulmonary lesions to the detected pathogen. Nonetheless, the gross lesions—cranioventral consolidation with focal abscessation—match those previously reported in *T. pyogenes*–associated pneumonia. Coupled with frequent isolation of *T. pyogenes*, the findings support its likely contribution to the observed lung disease. In addition, we did not test for bovine viral diarrhoea virus (BVDV), which impairs respiratory innate immune responses and predisposes calves to bacterial pneumonia (Al‐Haddawi et al. 2007). Accordingly, we cannot fully exclude a role for BVDV‐mediated immunosuppression in disease severity.

## 4 Conclusion

This case demonstrates that *T. pyogenes* can act as a major cause of pneumonia in calves. The detection of macrolide (*ermB* and *ermX*) and tetracycline (*tetW*) resistance genes highlights the emergence of AMR in *T. pyogenes*. To our knowledge, this is the first report to describe respiratory disease in calves primarily caused by *T. pyogenes* and to characterize AMR genes. These findings underscore the need for judicious antibiotic use and ongoing monitoring of resistance genes to prevent the spread of multidrug‐resistant bacteria in cattle populations.

## Author Contributions

**Min‐Jeong Ji**: investigation, experiments, data analysis, methodology, writing – original draft and editing. **Youngjun Kim**: sampling, clinical investigation, and primary clinician managing the case. **Byoung‐Soo Kim**: sampling and clinical investigation. **Jongho Kim**: pathologic interpretation. **Kyoung‐Seong Choi**: conceptualization, supervision, project administration, writing – review and editing. All authors read and approved the final manuscript.

## Funding

This work was supported by the National Research Foundation of Korea (NRF) grant funded by the Korea government (MSIT) (RS‐2025‐23525211).

## Ethics Statement

This study was exempt from ethical approval by the Institutional Animal Care and Use Committee (IACUC) of Kyungpook National University because the IACUC evaluates only laboratory animals housed in indoor facilities, not outdoor animals; therefore, ethics approval was not required.

## Conflicts of Interest

The authors declare no conflicts of interest.

## Data Availability

The data that support the findings of this study are available from the corresponding author upon reasonable request.
